# Proinflammatory Cytokines Correlate with Depression and Anxiety in Colorectal Cancer Patients

**DOI:** 10.1155/2014/739650

**Published:** 2014-09-17

**Authors:** Diego Oliveira Miranda, Taís Aparecida Soares de Lima, Lucas Ribeiro Azevedo, Omar Feres, José Joaquim Ribeiro da Rocha, Gabriela Pereira-da-Silva

**Affiliations:** ^1^Postgraduate Program in Public Health Nursing, University of São Paulo at Ribeirão Preto College of Nursing, 14040-902 Ribeirão Preto, SP, Brazil; ^2^University of São Paulo at Ribeirão Preto College of Nursing, 14040-902 Ribeirão Preto, SP, Brazil; ^3^Postgraduate Program in Genetics, Department of Biology, Institute of Bioscience, Language & Literature and Exact Science, São Paulo State University, 15054-010 São José do Rio Preto, SP, Brazil; ^4^Division of Coloproctology, Clinical Hospital, School of Medicine of Ribeirão Preto, University of São Paulo, 14048-900 Ribeirão Preto, SP, Brazil; ^5^Department of Maternal-Infant Nursing and Public Health, University of São Paulo at Ribeirão Preto College of Nursing, Avenida Bandeirantes 3900, 14040-902 Ribeirão Preto, SP, Brazil

## Abstract

The objective of this study was to investigate whether serum cytokine levels correlate with depression and anxiety in colorectal cancer (CRC) patients. Twenty patients hospitalized for surgical resection of CRC were included in the study group and twenty healthy volunteers comprised the control group. Depression and anxiety were analyzed using the Hospital Anxiety and Depression Scale (HADS), and serum levels of IL-1*β*, IL-6, IL-8, IL-10, IL-12, TNF-α, and TGF-*β* were measured by Cytometric Bead Array. We found that more than half of CRC patients presented clinically significant levels of anxiety or depression, and 65% of them manifested a combination of severe anxiety and depression. CRC patients had increased serum levels of IL-1*β*, IL-6, IL-8, and TNF-α but lower IL-10 concentrations. Correlation analysis between HADS score and cytokine levels revealed a positive association of anxiety and/or depression with IL-1*β*, IL-6, IL-8, and TNF-α and a negative correlation with IL-10. These results indicate that circulating proinflammatory cytokines are involved in the pathophysiology of anxiety and depression in CRC patients. A better understanding of the molecular mechanisms involved in these psychological disorders will allow the design of therapeutic interventions that lead to an improved quality of life and overall survival of CRC patients.

## 1. Introduction

Colorectal cancer (CRC) is the third most commonly diagnosed cancer in males and the second in females, with over 1.3 million new cases and 694,000 deaths estimated to have occurred in 2012, according to the World Health Organization [1]. In Brazil, the National Cancer Institute estimated that, in 2014, approximately 17,000 women will be affected by CRC, surpassing for the first time the number of cases of cervical cancer, trailing only breast tumors; 15,000 new cases are estimated among men, a number surpassed only by prostate and lung tumors [2].

Depression and anxiety are the most prevalent psychological disorders in patients with cancer, including CRC [3], occurring in approximately 30–40% of these patients [4, 5]. These symptoms impair the adherence to cancer treatment and patients' quality of life [6, 7]. The identification and proper management of these disorders is an important issue in oncology practice [8].

Evidence supporting a role of cytokines, especially IL-1, IL-6, IL-8, and TNF-α, in the pathophysiology of depression and anxiety, combined with the presence of high levels of these molecules in cancer patients, including CRC [9, 10], suggests that cytokines play a role in the etiology and pathophysiology of depression and anxiety in these subjects. In this study, we investigated whether serum cytokine levels correlate with anxiety and depression in CRC patients. A better understanding of the molecular mechanisms involved in these psychological disorders will allow the design of therapeutic interventions that lead to an improved quality of life and overall survival of CRC patients.

## 2. Material and Methods

### 2.1. Study Design and Patient Selection

Adult patients (*n* = 20) who were recently diagnosed (from 15 to 30 days) with colorectal cancer and admitted for tumor resection at the Division of Coloproctology of the Clinical Hospital of the Faculty of Medicine of Ribeirão Preto (HCFMRP/USP) were invited to participate in the study. The control group was composed of 20 healthy volunteers. Exclusion criteria were as follows: (a) individuals who previously received or are receiving radiotherapy or chemotherapy, (b) history of chronic inflammatory or autoimmune diseases, active infectious diseases, kidney disease, or diabetes, (c) use of immunosuppressive drugs, (d) patients diagnosed with schizoaffective disorder, bipolar disorder, or panic disorder, and (e) individuals with cognitive impairment that prevents them from understanding the study design and answering the HADS questionnaire. The study was approved by the Ethics Committee of the Ribeirão Preto College of Nursing, and all patients signed a consent form.

### 2.2. Measures

Depression and anxiety were measured using the Brazilian-Portuguese validated version [11] of the Hospital Anxiety and Depression Scale (HADS) [12]. It consists of 14 items and contains two subscales: anxiety and depression. Each item is rated on a four-point scale, giving maximum scores of 21 for both subscales. Scores of 11 up to 21 on each subscale are considered to be a significant case of psychological morbidity, while scores of 8–10 represent the “borderline” and 0–7 the normal [12]. For the combined anxiety and depression score (total HADS score), a cut-off score of 19 was used to identify patients with severe affective psychopathology [13]. Blood samples (8 mL) were collected from all study participants in the morning, at the same range of hours (more or less 1 hour difference) with vacuum tubes (Vacutainer-Becton Dickinson, Franklin Lakes, USA) and were allowed to clot for 1 hour ± 5 minutes at 37°C. After centrifugation at 1,000 ×g for 10 minutes at 4°C, the serum was stored at −80°C until use.

### 2.3. Cytokine Analysis

Serum concentrations of IL-1*β*, IL-6, IL-8, IL-10, IL-12, TGF-*β*, and TNF-α were measured by Cytometric Bead Array (CBA) kits according to the manufacturer's instructions (BD Biosciences, San Diego, USA).

### 2.4. Statistics

Student's *t*-test was used for comparing group means. The results were expressed as mean ± SD. Simple linear regression was used to verify the correlation between variables. The significance level used for the tests was 5%.

## 3. Results

### 3.1. Demographic Characteristics

Demographic characteristics of the study participants are shown in Table 1. The experimental group consisted of 20 patients of both genders (mean age of 63 years, SD = 11.8; 55% female). Most of them were married (80%) and had primary education (65%). Twenty healthy volunteers of both genders comprised the control group (mean age of 47.8 years, SD = 9; 60% female). Most of them were married (80%) and had secondary education (65%). We found no significant differences regarding age, gender, marital status, or education level between the groups.

### 3.2. HADS Score

Table 2 shows the scores of anxiety and depression obtained by the study participants. In the control group, all subjects (100%) showed no anxiety or depression, whereas 11 CRC patients (55%) presented clinically significant anxiety and 10 of them (50%) had depression symptoms. A combination of severe depression and anxiety, indicated by HADS total score >19, was found in 13 CRC patients (65%).

### 3.3. Serum Cytokine Levels

**Figure 1:**
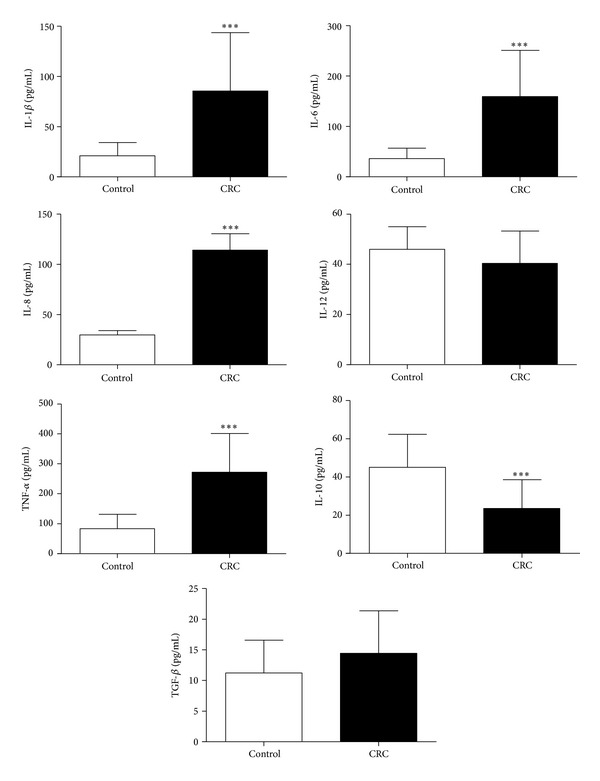
Serum concentrations of the cytokines IL-1-beta, IL-6, IL-8, TNF-alpha, IL-10, IL-12, and TGF-beta. Cytokine levels were measured by Cytometric Bead Array. Data represent mean ± SD; *n* = 20 for each group. ***P < 0.0001.

Compared to the control group, CRC patients had 3.2- to 4.4-fold higher concentrations of the proinflammatory cytokines IL-1*β* (mean: 85.5 versus 21.1 pg/mL), IL-6 (mean: 159.3 versus 36.1 pg/mL), IL-8 (mean: 114.3 versus 29.8 pg/mL), and TNF-α (mean: 272.4 versus 83.6 pg/mL). On the other hand, patients had lower (0.52-fold) IL-10 serum levels (mean: 23.5 versus 45.1 pg/mL) (*P* < 0.0001). No significant differences in IL-12 and TGF-*β* serum levels were observed between the groups (Figure 1).

### 3.4. Correlations between Cytokine Serum Levels and HADS Score

In CRC patients, anxiety, depression, and combined anxiety and depression were positively associated with IL-1 (*r* ^2^ = 0.48; *r* ^2^ = 0.49; *r* ^2^ = 0.59, resp.), IL-6 (*r* ^2^ = 0.49; *r* ^2^ = 0.36; *r* ^2^ = 0.49, resp.), IL-8 (*r* ^2^ = 0.62; *r* ^2^ = 0.44; *r* ^2^ = 0.65, resp.), and TNF-α (*r* ^2^ = 0.29; *r* ^2^ = 0.28; *r* ^2^ = 0.35, resp.) serum levels and negatively associated with IL-10 (*r* ^2^ = −0.36; *r* ^2^ = −0.25; *r* ^2^ = −0.21, resp.). No correlations between HADS score and cytokine serum levels were observed in the control group (Table 3).

## 4. Discussion

In this study, we showed that circulating proinflammatory cytokines are linked to depression and anxiety in CRC patients.

Depression and anxiety, which are considered the most important psychopathological comorbidities in CRC patients [3], are associated with poor outcomes, including decreased survival, reduced compliance with treatments, and an overall decreased quality of life [7]. Like previous studies, we found a high prevalence of depression and anxiety in CRC patients [4, 5, 14].

Although the occurrence of depression and anxiety may be related to many factors like therapy schemes and social and psychological characteristics [15], high levels of anxiety and depression among patients with CRC suggest the existence of a physiological mechanism, possibly directly related to the tumor, on the development of these comorbidities [16]. In advanced colorectal cancer, increased serum levels of the soluble portion of the interleukin 2 receptor alpha chain (sIL2rα) have been previously shown to correlate with HADS score, suggesting that tumor-induced immune activation contributes to depression in cancer [17]. The proposed “cytokine hypothesis” of depression/anxiety suggests that behavioral changes observed in cancer patients are caused by proinflammatory cytokines produced directly by cells in the tumor microenvironment, which influence multiple neuroendocrine pathways, altering mood severe enough to cause clinical depression and other comorbidities in cancer patients [14, 18–22]. This hypothesis is supported by studies showing a correlation between serum levels of proinflammatory cytokines and depressive symptoms in pancreatic and ovarian cancer patients [18, 23].

We found that CRC patients had increased serum levels of the proinflammatory cytokines IL-1, IL-6, IL-8, and TNF-α but lower IL-10 concentrations. In addition, the proinflammatory cytokines levels positively correlated with anxiety and/or depression in these patients, while IL-10 negatively associated with such psychological disorders. These results clearly suggest that proinflammatory cytokines are involved in the pathophysiology of depression and anxiety in CRC patients. In addition, they indicate that these disorders may not just be a reaction to a diagnosis of cancer but relate, at least in part, to immunological changes caused by the tumor itself.

Limitations of this study include its cross-sectional nature and the relative small number of colorectal patients studied. Therefore, we believe that further studies are required to confirm our results. Nevertheless, they have important clinical and research implications. Taken into account that CRC patients with high serum levels of proinflammatory cytokines may be at significant risk factor for developing anxiety and depression, cytokine levels may be used as a marker for the manifestation of such psychological disorders in these patients. Moreover, given that more than half of the CRC patients had clinically relevant levels of depression/anxiety and that individuals who are depressed and anxious are more likely to have health habits that put them at great risk, including worse sleep, a greater propensity for alcohol and drug abuse, worse nutrition, and less exercise [24], the results highlight the need of reducing the effect of psychological stress through social support. Thus, new therapeutic strategies to assist in alleviating symptoms in cancer patients might result from a better understanding of the role of cytokines in the pathophysiology of depression and anxiety in these subjects.

## Figures and Tables

**Table 1:** Demographic characteristics of study participants.

Characteristics	Control group *n* (%)	CRC patients *n* (%)	*P* value
Age (mean ± SD)	47.8 ± 9	63 ± 11.8	ns∗
Gender			
Male	8 (40)	9 (45)	ns^‡^
Female	12 (60)	11 (55)	
Marital status			
Single	2 (10)	1 (5)	ns^‡^
Married	16 (80)	16 (80)	
Divorced	1 (5)	1 (5)	
Widowed	1 (5)	2 (10)	
Education level			
Primary	6 (30)	13 (65)	ns^‡^
Secondary	13 (65)	6 (30)	
College/university	1 (5)	1 (5)	

*Student's *t*-test; ^‡^Pearson's chi-square test.

**Table 2:** Depression and anxiety score of study participants.

	Control group *n* (%)	CRC patients *n* (%)	*P* value
Anxiety			
0–7	17 (85)	3 (15)	<0.001^‡^
8–10	3 (15)	6 (30)	
11–21	0 (0)	11 (55)	
Mean ± SD	3.2 ± 2.7	11 ± 3.7	
Depression			
0–7	20 (100)	3 (15)	<0.001^‡^
8–10	0 (0)	7 (35)	
11–21	0 (0)	10 (50)	
Mean ± SD	2.5 ± 2.3	11 ± 3.4	
Depression and anxiety (total HADS score)			
≤19	20 (100)	7 (35)	<0.001^‡^
>19	0 (0)	13 (65)	

HADS: Hospital Anxiety and Depression Scale. ^‡^Pearson's chi-square test.

**Table 3:** Correlation between serum levels of cytokines and HADS score.

	IL-1	IL-6	IL-8	IL-10	IL-12	TNF-α	TGF-*β*
Control group							
Anxiety (HADS)	0.19	0.12	0.18	−0.14	0.11	0.15	−0.11
Depression (HADS)	0.12	0.13	0.12	−0.17	0.17	0.08	−0.12
D and A (total HADS)	0.12	0.14	0.22	−0.16	0.14	0.09	−0.12
CRC patients							
Anxiety (HADS)	0.48∗∗∗	0.49∗∗∗	0.62∗∗∗	−0.36∗∗	−0.14	0.29∗	−0.19
Depression (HADS)	0.49∗∗	0.36∗∗	0.44∗∗	−0.25∗	−0.02	0.28∗	−0.15
D and A (total HADS)	0.59∗∗∗	0.49∗∗∗	0.65∗∗∗	−0.21∗	−0.05	0.35∗∗	−0.11

HADS: Hospital Anxiety and Depression Scale.

D and A: depression and anxiety combined (total HADS).

Values represent the coefficient of determination (*r* ^2^) for simple linear regression.

Correlation is significant at ∗*P* < 0.05, ∗∗*P* < 0.001, and ∗∗∗*P* < 0.0001.
